# Spontaneous Extrapleural Hematoma in a Hemodialysis Patient Receiving Anticoagulation: A Rare Cause of Acute Chest Pain

**DOI:** 10.7759/cureus.109029

**Published:** 2026-05-17

**Authors:** El Mahdi Choukri, Zakaria Boulahcen, Nourelhouda Remok, Bouchra Dahmani, Siham Alaoui Rachidi

**Affiliations:** 1 Diagnostic and Interventional Radiology, Mohammed VI University Hospital, Tangier, MAR; 2 Radiology, Faculty of Medicine and Pharmacy, Abdelmalek Essaâdi University, Tangier, MAR

**Keywords:** anticoagulation, chest pain, computed tomography, dyspnea, extrapleural hematoma, hemodialysis, thoracic emergency

## Abstract

Extrapleural hematoma is a rare condition defined by blood accumulation between the parietal pleura and the endothoracic fascia. It is most commonly traumatic, whereas spontaneous cases remain exceptional. We report a case of a 60-year-old patient undergoing chronic hemodialysis and with documented anticoagulant exposure who presented with acute chest pain and dyspnea. Chest computed tomography (CT) revealed a right-sided extrapleural hematoma with characteristic imaging features, including lenticular morphology, spontaneous high attenuation on non-contrast CT, and displacement of extrapleural fat, associated with moderate right pleural effusion. Contrast-enhanced CT showed no active contrast extravasation. The patient was managed conservatively with a favorable clinical evolution. This case highlights the diagnostic value of CT in identifying extrapleural hematoma, distinguishing it from pleural collections, and guiding conservative management in clinically stable patients without evidence of active bleeding.

## Introduction

Extrapleural hematoma is an uncommon condition characterized by the accumulation of blood in the extrapleural space, located between the parietal pleura and the endothoracic fascia. Most reported cases occur in the setting of thoracic trauma, particularly rib fractures or blunt chest injury [1,2].

Computed tomography (CT) plays a central role in diagnosis by identifying the extrapleural location of the collection, its lenticular morphology, and its relationship with the chest wall and pleural space [3-5]. Because extrapleural hematoma may mimic hemothorax or pleural effusion on imaging, careful assessment of the extrapleural space is essential [6].

Although most published cases are traumatic, spontaneous extrapleural hematoma has also been reported, particularly in patients with bleeding risk factors such as anticoagulation or chronic hemodialysis [7,8]. The clinical presentation is often non-specific, most commonly including chest pain and dyspnea, which may delay diagnosis.

Early recognition is essential to avoid misdiagnosis and to guide appropriate management.

This case is educational because it illustrates the CT diagnosis of spontaneous extrapleural hematoma in a high-risk patient undergoing chronic hemodialysis and with documented anticoagulant exposure. It highlights the importance of recognizing characteristic extrapleural imaging features, particularly lenticular morphology and displacement of extrapleural fat, as well as the role of contrast-enhanced CT in assessing for active bleeding and supporting conservative management in a clinically stable patient.

## Case presentation

A 60-year-old patient undergoing chronic hemodialysis and with documented anticoagulant exposure was admitted to the emergency department for acute-onset chest pain associated with dyspnea. No history of thoracic trauma or recent invasive thoracic procedure was reported. The available medical record confirmed chronic hemodialysis and anticoagulant exposure; however, the exact indication for anticoagulation, anticoagulant dose, coagulation parameters, hemoglobin level, and dialysis vascular access were not documented. Therefore, the clinical context was interpreted as a spontaneous hemorrhagic event occurring in a patient with documented bleeding-risk factors, without implying that chronic hemodialysis itself necessarily required long-term anticoagulation.

Given the acute thoracic presentation and the patient’s increased bleeding risk, a chest CT was performed.

**Figure 1:**
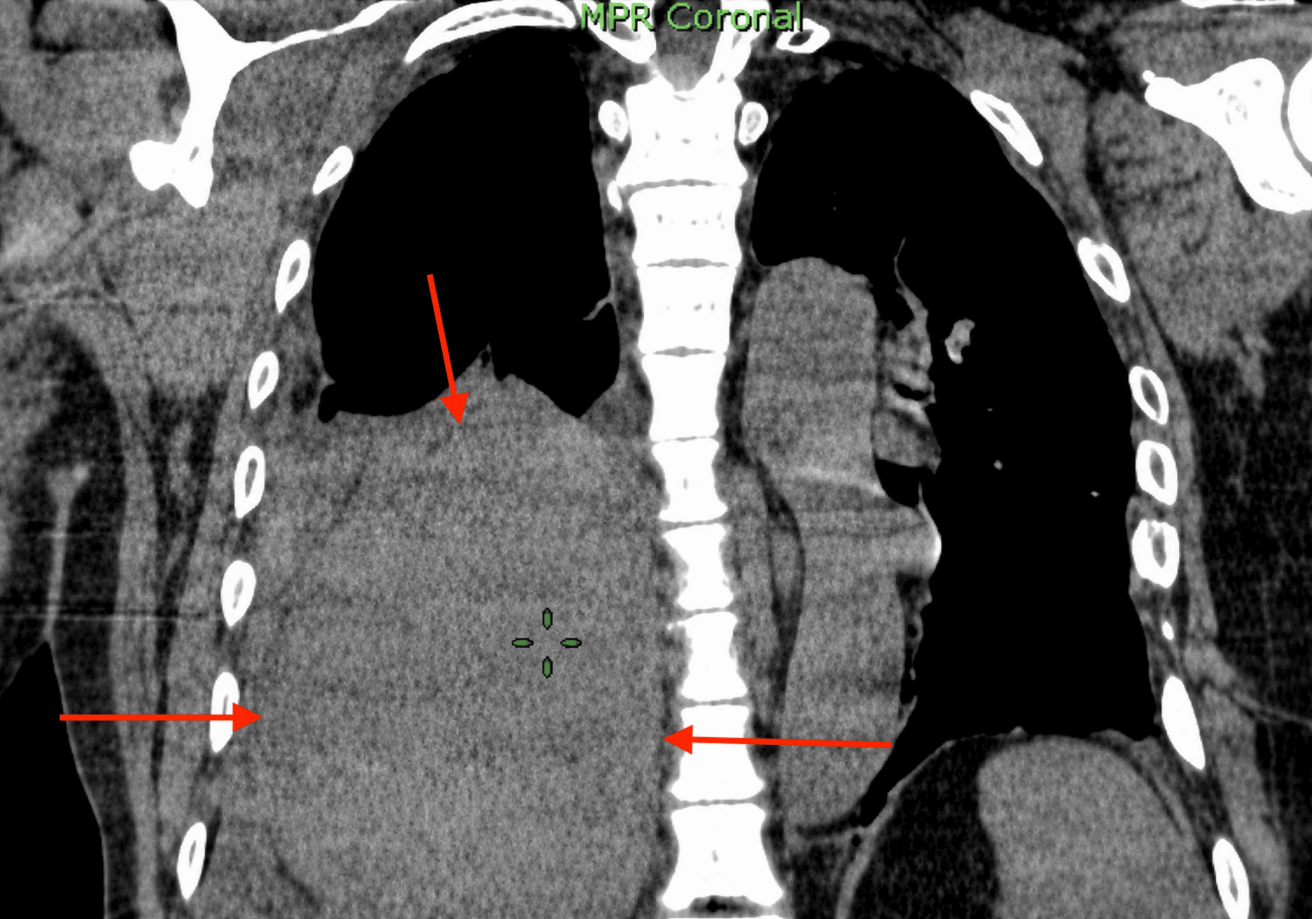
Coronal non-contrast CT image showing a right-sided peripheral extrapleural collection with lenticular morphology. The arrow indicates the extrapleural hematoma, and the downward arrow highlights displaced extrapleural fat, corresponding to the extrapleural fat sign.

Non-contrast chest CT demonstrated a right-sided peripheral thoracic collection with a lenticular, biconvex morphology along the chest wall (Figure 1).

**Figure 2:**
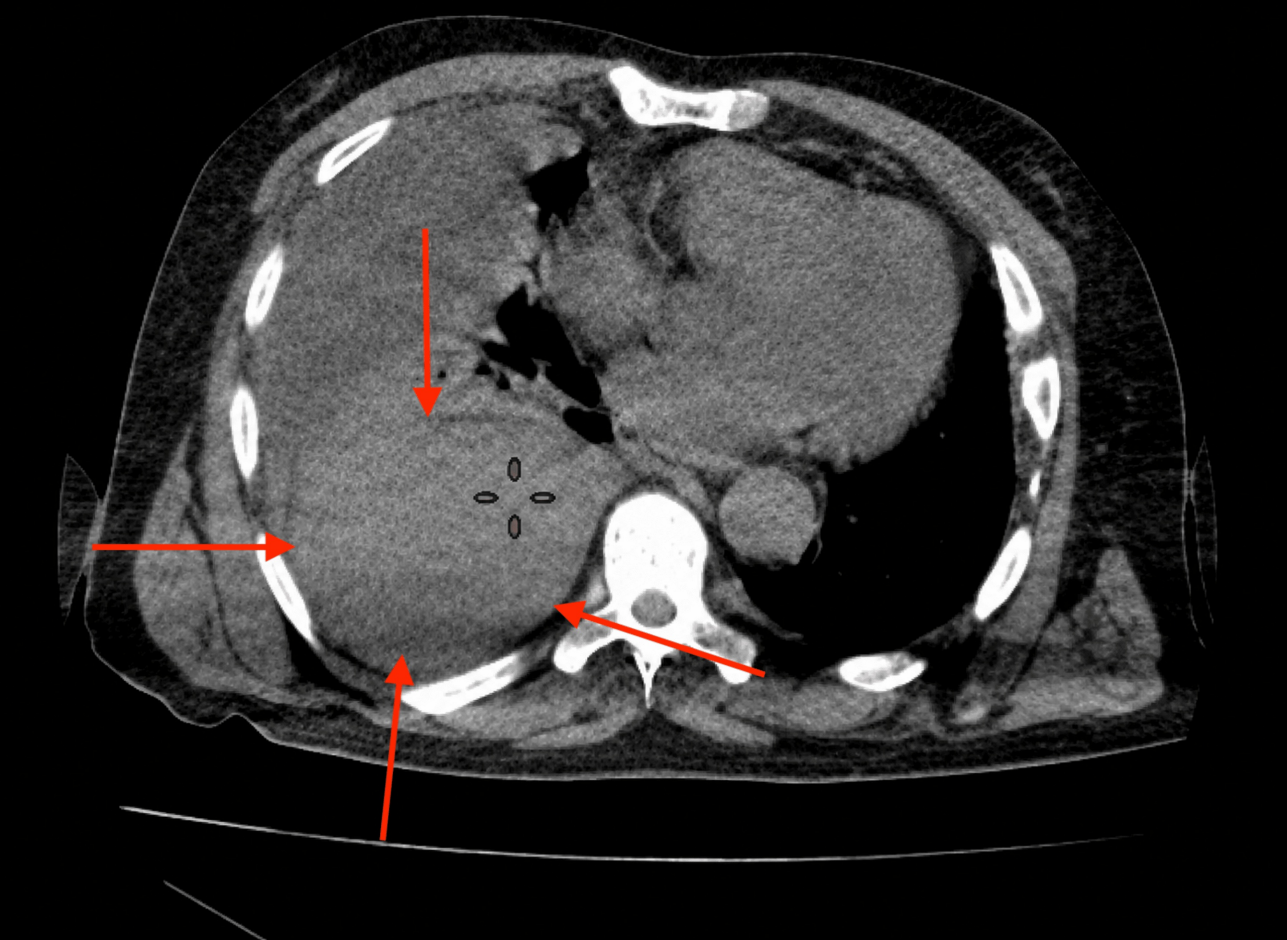
Coronal non-contrast CT image showing a right-sided peripheral extrapleural collection with lenticular morphology. The arrow indicates the extrapleural hematoma, and the downward arrow highlights displaced extrapleural fat, corresponding to the extrapleural fat sign.

The collection was spontaneously hyperdense, consistent with acute blood products, and was associated with a moderate right pleural effusion (Figure 2).

**Figure 3:**
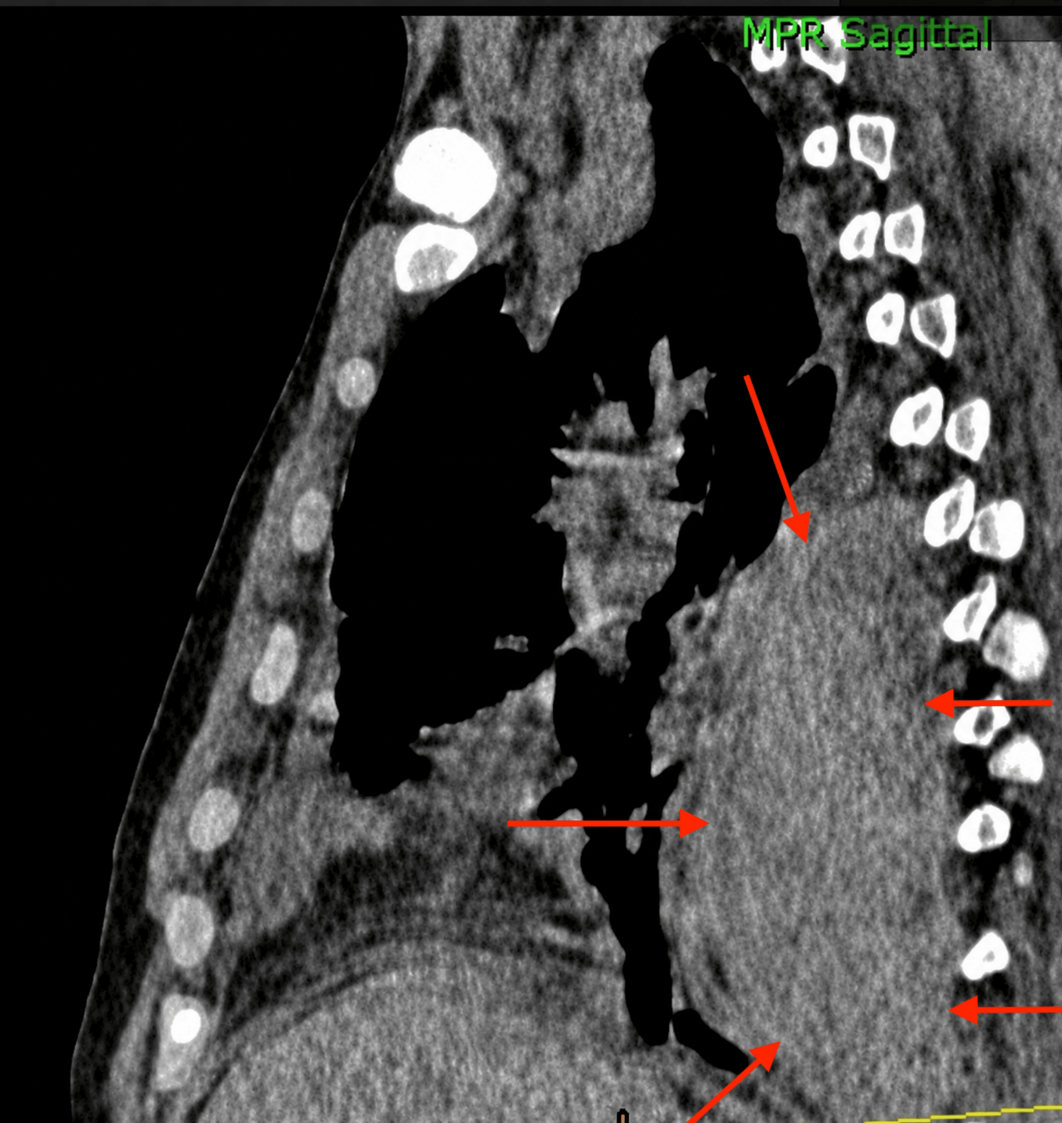
Sagittal non-contrast CT reconstruction showing extrapleural localization of the hematoma with compression of adjacent lung parenchyma.

Sagittal reconstruction confirmed the extrapleural location of the hematoma and showed compression of the adjacent lung parenchyma (Figure 3).

**Figure 4:**
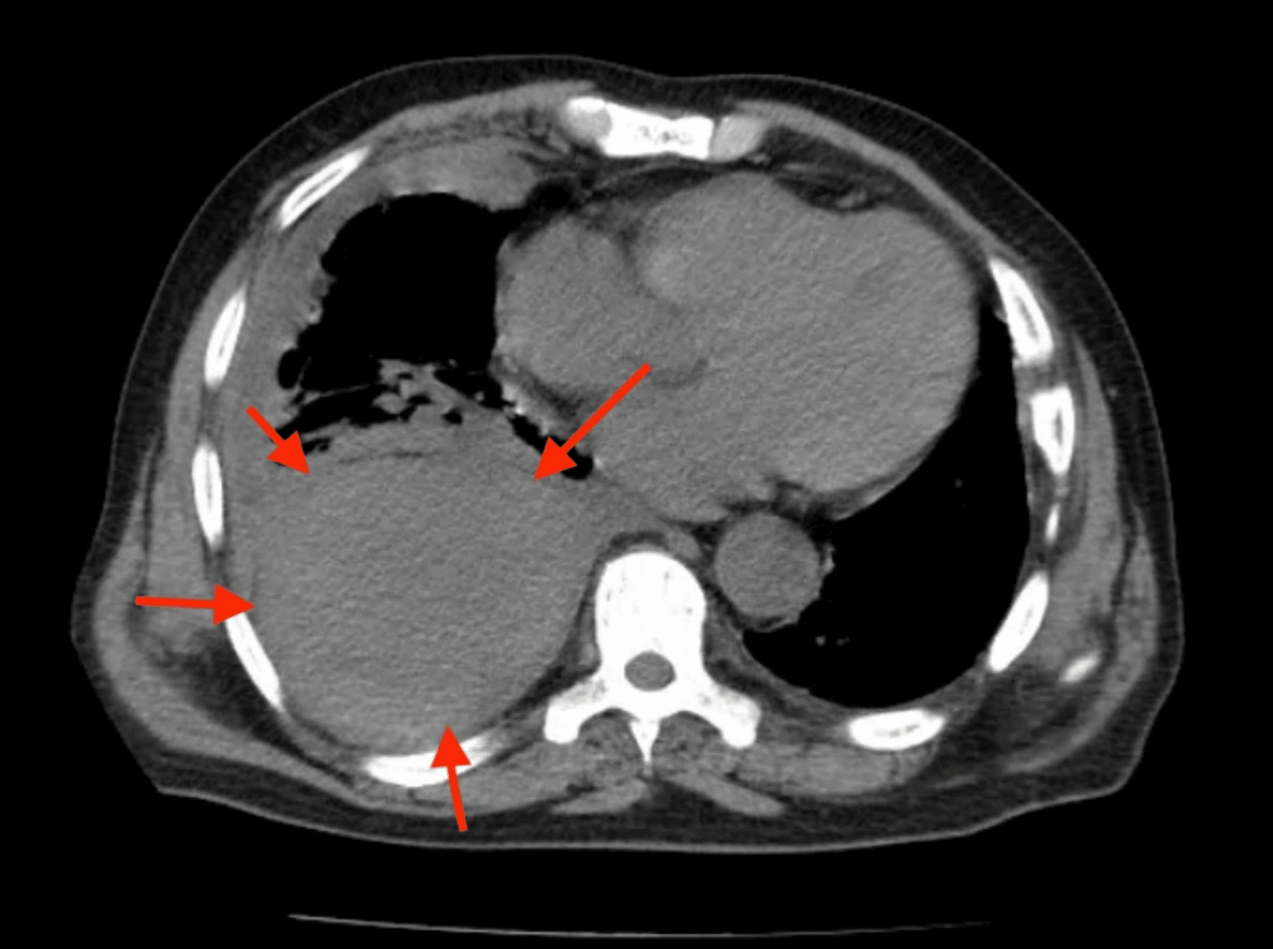
Contrast-enhanced axial CT image obtained with limited contrast opacification, showing no definite active contrast extravasation within the extrapleural hematoma. Moderate right pleural effusion is also present. The arrows indicate the extrapleural hematoma and associated pleural effusion.

Contrast-enhanced CT images, although limited by suboptimal contrast opacification, showed no definite active contrast extravasation within the extrapleural hematoma, with persistence of the associated moderate right pleural effusion (Figure 4).

Exact hematoma measurements and volumetric assessment of the pleural effusion were not documented in the available imaging report. Qualitatively, CT demonstrated a sizeable right-sided peripheral extrapleural collection with lenticular/biconvex morphology along the chest wall. The collection showed spontaneous high attenuation on non-contrast CT, consistent with acute blood products, and was associated with moderate right pleural effusion and compression of the adjacent lung parenchyma. Displacement of the extrapleural fat line was identified, supporting the extrapleural location of the hematoma. Contrast-enhanced CT was limited by suboptimal contrast opacification, but no definite active contrast extravasation was identified. No associated rib fractures were identified.

Based on the absence of trauma, documented anticoagulant exposure, chronic hemodialysis background, characteristic extrapleural CT morphology, displacement of the extrapleural fat line, and spontaneous high attenuation on non-contrast CT, the diagnosis of spontaneous extrapleural hematoma was retained.

Conservative management was selected because contrast-enhanced CT, although limited by suboptimal contrast opacification, showed no definite active contrast extravasation, and there was no immediate evidence of hemodynamic instability or progressive respiratory compromise in the available record. Detailed information regarding anticoagulation adjustment, transfusion requirement, and follow-up imaging was not documented in the available medical record. The clinical course was favorable, with no need for surgical drainage or endovascular intervention.

## Discussion

Extrapleural hematoma is an uncommon thoracic entity, most frequently described in the setting of trauma, particularly after rib fractures or blunt chest injury involving the chest wall or intercostal vessels [1,2]. Although most published cases are traumatic, spontaneous extrapleural hematoma has also been reported, especially in patients with bleeding risk factors such as anticoagulant therapy, coagulation abnormalities, or chronic hemodialysis.

Recognition of the extrapleural fat sign is particularly useful for differentiating extrapleural hematoma from hemothorax or loculated pleural fluid [3-6]. In an extrapleural hematoma, the collection develops outside the parietal pleura and may displace the extrapleural fat and parietal pleura inward, producing a lenticular or biconvex morphology along the chest wall [3-5]. In the present case, spontaneous high attenuation on non-contrast CT supported the presence of acute blood products, while contrast-enhanced CT, although limited by suboptimal contrast opacification, showed no definite active contrast extravasation. These imaging features, together with the absence of trauma and the patient’s bleeding-risk profile, supported the diagnosis of spontaneous extrapleural hematoma and favored conservative management [7-10].

Differentiating extrapleural hematoma from hemothorax is clinically important. Hemothorax occupies the pleural cavity and usually shows a dependent distribution, whereas extrapleural hematoma remains confined by the endothoracic fascia and typically maintains a lenticular configuration along the chest wall [6].

Spontaneous cases have been described in patients receiving anticoagulation and in patients undergoing chronic hemodialysis, supporting the role of bleeding risk factors in non-traumatic extrapleural hematoma [7,8]. In our case, the absence of thoracic trauma or recent invasive procedure, combined with chronic hemodialysis and anticoagulant therapy, supported the diagnosis of spontaneous extrapleural hematoma.

The clinical presentation is often non-specific. Chest pain and dyspnea are common symptoms and may initially suggest more frequent thoracic emergencies, including hemothorax, pleural effusion, pulmonary embolism, pneumonia, or empyema. Other imaging differentials include loculated pleural collections and pleural or chest wall tumors. Therefore, careful CT analysis is essential to avoid misdiagnosis.

CT is the imaging modality of choice for diagnosis and lesion characterization. Typical findings include a peripheral lenticular or biconvex collection, obtuse angles with the chest wall, displacement of extrapleural fat, and inward displacement of the parietal pleura [3-5]. Spontaneous hyperattenuation on non-contrast CT supports the presence of acute blood products, while contrast-enhanced CT is useful for assessing active bleeding.

In our case, CT demonstrated a right-sided extrapleural hematoma associated with moderate right pleural effusion (Figures 2, 4). This associated pleural effusion may increase diagnostic confusion with hemothorax or isolated pleural fluid. However, the lenticular morphology of the collection, its extrapleural location, and the absence of active contrast extravasation helped confirm the diagnosis.

Management depends on the patient’s hemodynamic status, respiratory impact, hematoma size, and evidence of ongoing bleeding. Stable patients without active extravasation may be managed conservatively with close clinical, laboratory, and radiological monitoring. In contrast, surgical or interventional treatment may be required in cases of hemodynamic instability, progressive enlargement, active bleeding, or significant respiratory compromise [9,10].

In our case, the patient was managed conservatively because contrast-enhanced CT, although limited by suboptimal contrast opacification, showed no definite active contrast extravasation, and there was no immediate evidence of hemodynamic instability or progressive respiratory compromise in the available record. The clinical evolution was favorable, with no need for surgical or interventional treatment.

This case has several limitations. Exact hematoma dimensions, volumetric assessment of the pleural effusion, detailed coagulation parameters, hemoglobin level, dialysis access information, anticoagulation indication, anticoagulant dose, and follow-up imaging were not documented in the available medical record. In addition, contrast-enhanced CT was limited by suboptimal contrast opacification. Nevertheless, the diagnosis was supported by the absence of trauma, the patient’s bleeding-risk profile, the characteristic lenticular extrapleural CT morphology, displacement of the extrapleural fat line, spontaneous high attenuation on non-contrast CT, and absence of definite active contrast extravasation within the limits of the available contrast-enhanced images.

## Conclusions

Spontaneous extrapleural hematoma is a rare but clinically important diagnosis to consider in patients presenting with acute chest pain and dyspnea, particularly in the presence of bleeding risk factors such as anticoagulant exposure and chronic hemodialysis.

CT plays a central role in diagnosis by accurately localizing the hematoma within the extrapleural space, identifying key imaging features such as lenticular morphology and displacement of extrapleural fat, assessing associated pleural effusion and lung compression, and evaluating for active bleeding. In this case, the absence of trauma, characteristic extrapleural CT morphology, spontaneous high attenuation on non-contrast CT, displacement of the extrapleural fat line, and absence of definite active contrast extravasation within the limits of suboptimal contrast opacification supported conservative management, with favorable clinical evolution.
